# Current Analytical Methods and Challenges for the Clinical Diagnosis of Invasive Pulmonary Aspergillosis Infection

**DOI:** 10.3390/jof10120829

**Published:** 2024-11-28

**Authors:** Madeline C. R. Schwarz, Alex E. Moskaluk, Joshua B. Daniels, Sue VandeWoude, Melissa M. Reynolds

**Affiliations:** 1 Department of Chemistry, Colorado State University, 1801 Campus Delivery, Fort Collins, CO 80523, USA; madeline.roach@colostate.edu; 2 Department of Microbiology, Immunology, and Pathology, Colorado State University, 1619 Campus Delivery, Fort Collins, CO 80523, USA; alex.moskaluk@uoguelph.ca (A.E.M.); josh.daniels@colostate.edu (J.B.D.); sue.vandewoude@colostate.edu (S.V.); 3 Department of Pathobiology, University of Guelph, 50 Stone Road East, Guelph, ON N1G2W1, Canada; 4 Department of Chemical and Biological Engineering, Colorado State University, 1370 Campus Delivery, Fort Collins, CO 80523, USA; 5 School of Biomedical Engineering, Colorado State University, 1376 Campus Delivery, Fort Collins, CO 80523, USA

**Keywords:** invasive pulmonary aspergillosis, diagnosis, polymerase chain reaction, peptide nucleic acid–fluorescence in situ hybridization, enzyme-linked immunosorbent assay, high-pressure liquid chromatography mass spectrometry

## Abstract

In the last decade, pulmonary fungal infections such as invasive pulmonary aspergillosis (IPA) have increased in incidence due to the increased number of immunocompromised individuals. This increase is especially problematic when considering mortality rates associated with IPA are upwards of 70%. This high mortality rate is due to, in part, the length of time it takes to diagnose a patient with IPA. When diagnosed early, mortality rates of IPA decrease by as much as 30%. In this review, we discuss current technologies employed in both medical and research laboratories to diagnose IPA, including culture, imaging, polymerase chain reaction, peptide nucleic acid–fluorescence in situ hybridization, enzyme-linked immunosorbent assay, lateral flow assay, and liquid chromatography mass spectrometry. For each technique, we discuss both promising results and potential areas for improvement that would lead to decreased diagnosis time for patients suspected of contracting IPA. Further study into methods that offer increased speed and both analytical and clinical sensitivity to decrease diagnosis time for IPA is warranted.

## 1. Introduction

Humans are exposed to fungal spores daily. Globally, the average abundance of spores in the air ranges between 10^3^ and 10^4^ spores per cubic meter [1]. Infections from airborne fungal spores mainly occur invasively [2]. Despite their low occurrence rates, a study performed in 2017 estimated that fungal infections result in the death of more than 1.5 million people per year. This is similar in scale to the mortality rate of tuberculosis, and three times greater than that of malaria [3]. One common disease-causing genus of fungi is *Aspergillus*. *Aspergillus* is often associated with spoilage and mycotoxin production in food, but is also a cause of an array of infectious and allergic human and animal diseases [4]. In this review, we discuss *Aspergillus*, including the associated human diseases and diagnostic techniques available, with a focus on diagnosis of pulmonary aspergillosis.

### 1.1. Aspergillus

*Aspergillus* belongs to the *Trichocomaceae* family under the order *Eurotiales* and phylum *Ascomycota*. The genus *Aspergillus* contains more than 250 species grouped into eight subgenera and species complexes [5]. The subgenera include *Aspergillus*, *Circumdati*, *Cremei*, *Fumigati*, *Nidulantes*, and *Polypaecilum* [6]. Important and medically relevant species complexes include *Aspergillus fumigatus*, *Aspergillus flavus*, *Aspergillus terreus*, *Aspergillus niger*, *Aspergillus nidulans*, and *Aspergillus ustus* [5]. *Aspergillus* was first described in 1729 by Micheli, who named it for the brush used for sprinkling holy water in churches [5,7]. Aspergillosis, the name for the invasive infection by *Aspergillus* sp., was first described by Reaumur in 1749 in birds. The pathogenic *Aspergillus candidus* was detected in an air sac lesion of a bullfinch by Rayer and Montagene in 1842 [5]. The first time “Aspergillosis” was used to describe a respiratory disease was in 1863, when *A. fumigatus* was detected in the lung of a great bustard [5]. The first description of a human disease caused by *Aspergillus* sp. was recorded by Sluyter in 1847 [7].

**Figure 1:**
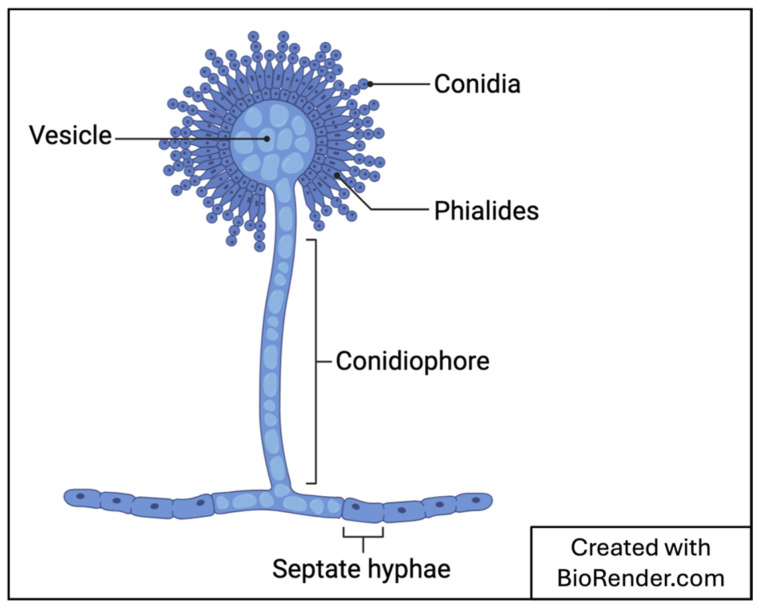
Generic structure of *Aspergillosis* sp.

*Aspergillus* is a widespread fungal genus that has both pathogenic and beneficial species. It is commonly found in soil, air, water, vegetables, and feed as saprophytes [5,8]. Due to its concentration in air (between 0.2 and 15 conidia/m^3^ and up to 10^7^ conidia/m^3^ in some settings), *Aspergillus* can be considered a common laboratory contaminant [5]. Some species can be identified based on their microscopic morphological appearances. Generally, and as shown in Figure 1, its mycelia are composed of septate hyphae with dichotomous branching. The primary branch that originates from the vegetative portion of the thallus is called the “foot cell”. This is branched into conidiophores that contain vesicles, the shape of which is dependent on the species. Parts of the vesicles are covered with series of phialide that are arranged in various ways. Asexual spores (conidia) originate from these phialides. The part of the vesicle covered in phialide, number of series of phialide, and the phialide’s arrangement on the vesicle are entirely dependent on the species of *Aspergillus*. In tissue samples, only the mycelia are observed, while conidiophores are sometimes found in body cavities that contain air [5].

*Aspergillus* can be isolated on Sabouraud dextrose agar (SDA) or Czapek Dox agar (CDA), among others, to observe visual characteristics between the major human pathogens [5,9]. *A. fumigatus* colonies are a white cottony mold, which becomes velvety or granular with green coloration after several days. *A. niger* colonies are white in color when they begin to grow, but later become black as growth continues. *A. terreus* colonies start white as well, but later become cinnamon colored with a “sugary texture” [5]. *A. flavus* has spreading yellow-green colonies that have phialides over their entire surface, while *A. nidulans* has a brown pigment that helps it blend in with the soil where it commonly grows [9].

### 1.2. Diseases Caused by Aspergillus Species

*Aspergillus* species are associated with a spectrum of respiratory diseases in both humans and animals, affecting areas including nasal cavities, sinuses, airways, and lung tissues and cavities. The diseases associated with *Aspergillus* range from allergies of variable severity to invasive infections with high mortality rates in humans [10]. Rates of infection and mortality are elevated in immunocompromised individuals and patients on immunosuppressive therapies [11,12,13]. *Aspergillus*-induced diseases can be broadly classified according to their characteristics as chronic colonization, allergic diseases, and invasive diseases. It is important to note, however, that any given clinical condition may fall into two or more classifications, rather than just one [7,9,14].

#### 1.2.1. Chronic Colonization

Chronic colonizations are broadly classified as fungal infections that do not cause tissue damage, meaning they grow inside the body but do not directly damage tissue. Bronchopulmonary colonization refers to the infestation of airways, but without invasion of tissue. In its most uncomplicated form, this colonization is not technically an infection. However, chronic colonization has been shown to have a high incidence in patients with other pulmonary infections such as chronic bronchitis, primary ciliary dyskinesia syndrome, and cystic fibrosis. Some patients suffer from adverse immune system effects from *Aspergillus* antigens, such as skin reactivity [7,14].

An *Aspergillus*-specific chronic colonization is called an aspergilloma. Also known as a fungus ball, aspergillomas are proliferations of the fungus in a poorly drained lung space. If an intrathoracic cavity communicates with the bronchial tree and does not drain, it can become colonized by an *Aspergillus* species and the colony can grow for months or years. Patients who have contracted pulmonary tuberculosis with healed cavities and chronic pulmonary sarcoidosis with cystic spaces are prime breeding grounds for fungal balls [7,9,10,14].

#### 1.2.2. Allergic Disease

*Aspergillus* species can also contribute to a variety of allergic diseases including allergic asthma, allergic rhinitis, allergic sinusitis, extrinsic allergic alveolitis (also called hypersensitivity pneumonitis (HP)), and allergic bronchopulmonary aspergillosis (ABPA). Most allergic airway diseases are managed in general practice and testing for fungus as an etiology is not common until the disease progresses [14,15].

Allergic asthma induced by fungal spores is characterized by higher responsiveness of the tracheobronchial tree to stimuli, potentially causing chronic respiratory symptoms of variable severity, which can lead to patients requiring corticosteroids [15]. Interestingly, corticosteroid administration can be a contributing factor for fungal infections in immunocompromised individuals [16]. Patients with mold sensitivities, like those with compromised immune systems, develop asthma slowly but persistently and have other symptoms similar to a cold followed by coughing and wheezing [7,10].

Allergic rhinitis describes a nasal allergy and is commonly called hay fever. This condition develops because of the interactions of allergens, like molds, with immunoglobulin E antibodies on the nasal mucosa epithelium [7,10].

Allergic sinusitis, or sinus disease, includes fungal ball production and allergic fungal sinusitis. The fungal balls produced differ considerably from aspergillomas as the fungus does not invade tissues but chronically affects multiple sinuses [7,10].

HP is caused by the exposure to multiple organisms by inhalation. *Aspergillus* species are commonly associated with HP. If chronic HP goes untreated, it can cause irreversible lung damage. Continuous exposure to the antigen causing HP can result in an intense inflammatory reaction in the lungs [7,10].

ABPA is one of the most commonly contracted diseases associated with *Aspergillus*, usually by asthmatic patients in the hospital and rhinitis and/or cystic fibrosis patients. ABPA is the result of hypersensitivity to *Aspergillus* antigens in patients with long-standing asthma triggered by an allergen. While ABPA begins as an easily reversible asthmatic syndrome, it can progress to a more difficult-to-manage asthmatic state with any substance denser than air, such as spores from *Aspergillus*, lingering in the parenchyma of the lungs. A strong sensitivity to the antigen produced by the body when *Aspergillus* is present can also develop, leading to a diagnosis of ABPA [7,10,14].

#### 1.2.3. Invasive Diseases

The final classification for conditions caused by *Aspergillus* species is invasive diseases. Invasive pulmonary aspergillosis (IPA) is primarily an infection of immunocompromised patients but can also occur in patients with chronic diseases like diabetes and some cancers or as a postoperative complication. IPA can also evolve into a superinfection following antibiotic therapy [7,8,14,17,18]. In the last decades, the incidence of IPA has increased due to an upsurge in immunocompromised patients [19]. IPA also has an associated mortality rate of 70% if diagnosis is delayed, making it the deadliest form of disease caused by *Aspergillus* species [20,21]. However, with early diagnosis, mortality rates for IPA may decrease to as low as 30% [20]. Due to this, the development of fast and accurate detection methods for this disease is vital for improving patient outcomes. Current clinical detection methods for IPA along with methods in the research stage will be the focus of the remainder of this work.

## 2. Current Detection Methods for Pulmonary Aspergillosis

### 2.1. Clinical Specimens

If a patient is suspected of having a fungal pulmonary infection, several sample types can be collected. The diagnostic standard for IPA is culture of tissue, which is collected using either fine-needle aspiration or open biopsy [10]. In the absence of tissue specimens, blood samples may be collected for culture, though results are rarely positive when using blood culture, or other testing for DNA or biomarkers. Fluid samples may also be collected from the lower respiratory tract using a collection technique called bronchoalveolar lavage (BAL) [5,8,22]. Other non-sterile samples, like sputum, can also be examined [8,22,23,24].

### 2.2. Fungal Culture

The most basic and primary method for diagnosing IPA is fungal culture. Fungal culture has been the standard for detection of IPA since definitions were proposed by the European Organization for the Research and Treatment of Cancer/Mycoses Study Group (EORTC/MSG) [25]. When taken from a sterile site, a positive culture provides proven diagnosis of IPA [25,26]. Culture allows for species identification and antifungal susceptibility testing, which allows for targeted therapy [25,26].

While culture is the most used and often the first step in diagnosis, it has several limitations. First, it is recommended that samples thought to contain fungus should be cultured for at least 7 days, delaying the time to diagnosis [23]. Culture is also a relatively insensitive method, with positivity rates ranging from 11.8% to 81.0% depending on the study and the form of aspergillosis (IPA vs. chronic colonization) studied [21,22,23,27,28,29,30,31]. Cultures also require specialized knowledge and experience to determine the identity of the colonies [19,31]. Growth of *Aspergillus* sp. in culture does not also always equate to a diagnosis of IPA, as the type of clinical sample used and the ubiquity of fungal spores lead to frequent contaminations during sample processing [17,18,32,33].

### 2.3. Imaging, Direct Microscopic Examination, and Histopathology

Imaging is another common method recognized by the EORTC/MSG that aids in the diagnosis of IPA. The early stages of IPA are often inconspicuous upon conventional thoracic radiography (X-ray), making it a suboptimal diagnostic tool [34]. The most common method of lung imaging for clinical purposes is not X-ray, however, it is high-resolution computed tomography (HRCT) [26,35]. A prevalent marker for IPA in HRCT is the “halo sign”. IPA is the most common cause of the halo sign in patients at high risk for fungal infections [8,17,18,19]. The halo sign is not, however, exclusive to IPA, as it can be linked to other infectious agents, bleeding, and leukemic infiltrates [23,36]. Using the halo sign as a marker does not afford a short time to diagnosis, as it does not appear until the third week of infection [37]. Other signs of IPA in HRCT include the air crescent sign, multiple pulmonary nodules, bronchopneumonia, consolidation, cavitation, plural effusions, ground-glass opacities, tree-in-bud opacities, and atelectasis. However, these signs are all nonspecific and do not only indicate IPA [35]. For example, tree-in-bud opacities can also be found in *Mycobacterium* infections and other non-infectious conditions [16,18,22,37]. Furthermore, another imaging modality that can be utilized for IPA is magnetic resonance imaging (MRI). MRI of the lungs is challenging due to the lack of detectable protons in air-filled spaces, so it has received low amounts of attention as a diagnostic imaging tool for IPA [38]. Despite this, studies have been performed to evaluate MRI as a diagnostic tool. One study performed in vivo on mice showed that lung lesions due to IPA could be visualized and quantified using MRI [39]. This study, while promising, has not been replicated using human patients, and requires advanced MRI techniques that are not broadly available outside of research settings.

Histopathology of tissue specimens is another diagnostic technique used for IPA, often in tandem with imaging like HRCT [40,41,42]. The EORTC/MSG guidelines state that a histopathologic examination of sterile material can lead to a confirmed diagnosis of IPA [26]. Signs of IPA infection include areas of tissue necrosis where hyphae are aligned in a radial pattern, fusion of solidified lobules with a necrotic cavity in the center, infarction, and/or embolism [41]. The main drawback of histopathology is the specialized knowledge and experience needed to determine infection.

Direct microscopic examination is an alternate technique recognized by the EORTC/MSG guidelines to confirm a diagnosis of IPA [26]. Direct examination involves microscopically examining samples for the fungal elements of *Aspergillus* sp., often with a stain or dye [8,19,43]. While direct microscopic examination can directly determine an infection, the technique cannot definitively distinguish *Aspergillus* sp. from other filamentous fungi and is not as sensitive as other standard techniques (41.8%) [44,45].

While culture, imaging, direct microscopic examination, and histopathology are the most common modalities for detection and diagnosis of IPA, all have limitations, primarily that culture, imaging, and direct examination are relatively insensitive, and that obtaining specimens for histopathology is invasive. More sensitive methods have been developed which decrease the time needed to obtain a diagnosis and are arguably less invasive.

### 2.4. PCR

The polymerase chain reaction (PCR) is a technique used to amplify small segments of DNA. PCR has been used to identify pathogenic fungi in a variety of samples since the late 1990s [8,17,46]. Kappe et al. (1998) described one of the first techniques using PCR to identify *A. fumigatus* and *A. flavus*, among other fungal species, in human tissues. They used a targeted area of 18S rDNA, with primers designed based on sequence conservation among fungi with concurrent dissimilarity to other eukaryotic sequences. This region can be successfully amplified from many medically relevant fungal agents [47]. A study performed by Kawamura et al. (1999) tested serum samples using an 18S-based nested PCR test. They showed that, in comparison to two antibody/antigen assays, the nested PCR was the most sensitive of the three techniques, with a clinical sensitivity of 89% and selectivity of 100% [48]. It is important to note that the sensitivity and selectivity reported in Kawamura et al. were obtained from a combination of samples from patients with varying forms of aspergillosis, including chronic pulmonary aspergillosis and IPA, and the form of aspergillosis can affect the sensitivity and selectivity of a diagnostic method. Kami et al. (2001) published the first report on the use of an 18S-based quantitative real-time PCR (qPCR) for the diagnosis of IPA [49]. In their work, DNA was extracted from both whole blood and plasma for their testing, as well as from pathogenic fungal species like *A. fumigatus*, *A. niger*, and *A. terreus*. The samples were also run using ELISA and (1→3)-β-_D_-glucan to compare clinical sensitivity and specificity. The qPCR test had the highest clinical sensitivity of the three tests, at 79%, and the highest clinical specificity at 92% [42]. The lower clinical sensitivity reported by Kami et al. contributed to false-positive results from control patients, and false positives in qPCR occurred more frequently than in the other tests in the study [49]. Kami et al. opined that the false positives were likely not due to environmental contamination, but rather “…*Aspergillus*-specific DNA might have existed in these samples” [49]. Since these first tests, numerous studies on qPCR for the diagnosis of IPA have been performed, and several reviews detailing the advancements in the technique have been written [50,51,52]. Due to these advancements and the extensive studies performed on qPCR for detection of IPA, the EORTC/MSG added qPCR testing as a diagnostic tool for IPA in 2020, along with publishing diagnostic criteria for plasma, serum, whole blood, and BAL fluid [26]. Table 1 describes the specificity and sensitivity of various PCR assays summarized by White et al. (2015) [50].

Despite being added as a viable technique for diagnosis of IPA, there are still limitations to PCR and qPCR. There is a high diversity of PCR assays, leading to a lack of protocol standardization. This was the largest factor that limited the use of the technique in hospital routines [58]. The diversity in assays comes from the primers, the amount of sample that needs to be analyzed, and whether the assay performs basic PCR or qPCR. The method of DNA extraction is also a factor in the performance of the assays. Another limitation to qPCR is the signal produced by active infections. The signal produced in active infections also falls within the range for signals attributed to antifungal therapy in both experimental models and clinical contexts [49]. Antifungal agents used for therapies in patients could account for some of the false-negative qPCR results reported [31]. To combat the assay diversity, false negatives, and quantitative issues with qPCR, more work needs to be carried out to improve the technique.

### 2.5. ELISA

**Figure 2:**
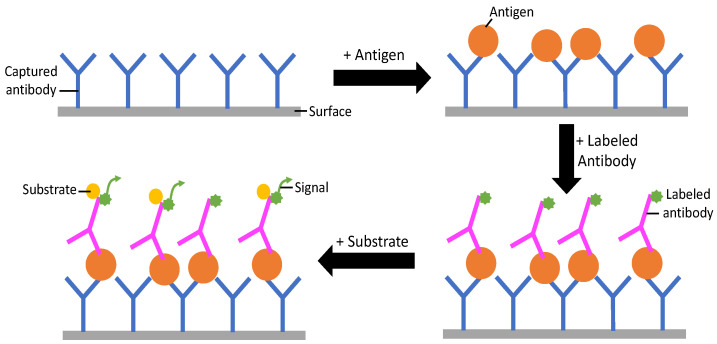
General scheme for sandwich ELISA signal detections—an antigen is added to an antibody captured on the surface of the ELISA. Then, a labeled antibody is added. When the molecule of interest (substrate) is added to the labeled antibody, a signal is produced.

Enzyme-linked immunosorbent assay (ELISA) is an assay technique designed to detect and quantify a soluble substance, such as peptides, proteins, antibodies, or antigens. Specifically, when the target of an ELISA is an antigen, the test is known as a biomarker assay. Galactomannan is a heat-stable heteropolysaccharide that is present in *Aspergillus* sp. cell walls [31]. Heteropolysaccharides are polysaccharides that contain two or more different monosaccharide units [59]. Detection of galactomannan molecules is achieved using a sandwich ELISA, shown in Figure 2, which is possible due to the multiple immunoreactive sites where antibodies can attach on a single molecule. The EORTC/MGS has determined that the PlateliaTM assay (Bio-Rad Laboratories, Marnes-la-coquette, France) [60], and other commercially available assays used to detect galactomannan (GM tests), are acceptable tools for diagnosis of IPA and have published diagnostic criteria for serum, plasma, BAL fluid, and cerebral spinal fluid [26]. GM tests are often used in conjunction with PCR to diagnose IPA. GM tests have a lower specificity than expected, ranging from 85 to 96% [61,62,63]. This is likely due to some form of cross reactivity in the ELISA, but it is unclear whether the lowered specificity comes from exogeneous galactomannan or other molecules that also react with the antibody used. The clinical sensitivity of the galactomannan ELISA is variable, with a range of 29–100% [8,31,48,61,62,63]. Another molecule available for use in ELISA testing for IPA is Beta-(1,3)-D-glucan (BDG). BDG is also a component in *Aspergillus* sp. cell walls, and the EORTC/MGS has determined the Fungitell test (Associates of Cape Cod, Falmouth, MA, USA) to be useful in specific clinical settings [8,26]. These include individuals with neutropenia following hematopoietic stem cell transplants, patients in the ICU, and patients with hematologic malignancies [26]. Studies reporting BDG tests show a lowered variance for clinical sensitivities, between 85 and 90%, when compared to GM tests, but the selectivity for BDG assays is much lower, between 26 and 85% [64,65,66]. The variability reported for fungal ELISA could be due to a variety of factors, including the patient group, antifungal therapy, and differences in sampling strategies. Studies show that the sensitivity for detection in profoundly immunocompromised patients is in excess of 90%, while, in other settings (such as patients undergoing antifungal therapy), sensitivity is lower. One important drawback of BDG tests specifically is that BDG is also found in the cell walls of *Penicillium* and other fungal species, which can cause sample contamination and false-positive results [64]. Finally, there has been no optimal sampling strategy defined for the detection of IPA, which could affect the clinical sensitivity of the technique.

### 2.6. Peptide Nucleic Acid–Fluorescence In Situ Hybridization (PNA-FISH)

**Figure 3:**
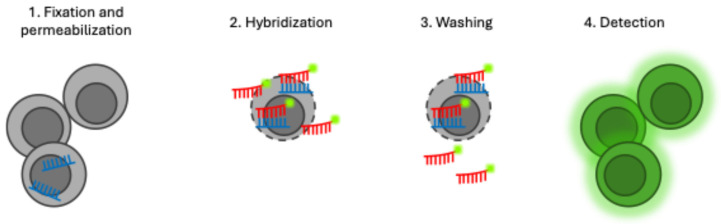
General schematic for PNA-FISH. First, the cell undergoes permeabilization and the RNA is fixated. Second, the target RNA is hybridized with the PNA probe. Third, excess probes are washed from the cells. Finally, the cells are detected using epifluorescence microscopy.

Fluorescence in situ hybridization (FISH) is a molecular technique that uses single-cell identification to detect microorganisms rapidly and directly in environmental and medical samples [67,68]. FISH can be divided into four main steps, shown in Figure 3: fixation and permeabilization of the sample, hybridization of the target RNA with the fluorescent probe, a washing step where unhybridized probes are washed from the sample, and detection of the target sequence using epifluorescence microscopy [67,69]. Synthetic nucleic acid mimics (NAMs) have been developed for clinical samples using a peptide nucleic acid (PNA). In PNAs, the negatively charged sugar–phosphate backbone of DNA is replaced by a neutral polyamide backbone. PNA molecules are also neutral, leading to no electrostatic repulsion between the PNA and the negatively charged sugar–phosphate backbone of the target molecule. This allows a stronger bond to the target sequence, and greater thermal stability of PNA/DNA duplexes when compared to DNA/DNA or DNA/RNA duplexes [67]. PNA-FISH protocols are relatively short (60–90 min time to result), no pre-treatment of the sample is required, and it is not necessary to extract DNA from the sample. This method also allows identification at the genus and species level [70,71]. The main limitation of PNA-FISH is the requirement of an organism concentration that is, at a minimum, between 10^2^ and 10^5^ colony-forming units/mL for detection [70]. These levels are much higher than expected in the early stages of IPA infection. Other microbiota in the sample may also exhibit autofluorescence, which decreases the signal-to-noise ratio and can mask some fluorescent signals [19].

PNA-FISH was first developed to test for filamentous fungi, including Aspergillus niger, in 2004 [72]. In 2020, a kit was developed by Biomode 2, SA, in Portugal and tested on a variety of clinical samples using PNA-FISH to detect Aspergillus fumigatus [70]. Reported total time to result was approximately 25.5 h for clinical samples (including the 24 h pre-enrichment step) with a specificity of 100%. With a clinical sensitivity of 79%, a lingering challenge with this technique is the persistence of false-negative results [70]. PNA-FISH also relies on specialized microscopy, epifluorescence microscopy, that requires not only specialty equipment but also expert visualization. PNA-FISH is currently not used in medical laboratories due to these issues, but, with further testing, shows promise as a detection method for IPA.

### 2.7. Lateral Flow Assays

Lateral flow assays (LFAs) are paper-based tests used widely in hospitals, physician’s offices, and clinical laboratories. A variety of sample types can be tested using LFAs, including urine, saliva, sweat, blood, and other fluids. LFAs are good candidates for new assays due to their low cost of production, ease of use, and rapid time to results, which can be as quick as 15 min [32,73]. *Aspergillus*-specific LFAs are a relatively new technology. One assay, developed by the University of Exeter, uses the monoclonal antibody JF5 to bind to a protein epitope present on an antigen secreted during the active growth of *A. fumigatus* [32]. Other LFAs, such as ELISA biomarker assays, target a specific antigen like galactomannan. As shown in Table 2, early trials and studies show a wide variety of sensitivity and selectivity [74]. The effectiveness of LFAs to date in clinical diagnosis is still limited, however, and more testing is needed on current assays.

### 2.8. LC-MS

Combination high-performance liquid chromatography and mass spectrometry (LC-MS) allows for high analytical specificity and sensitivity. LC-MS, and its tandem mass spectrometry counterpart (LC-MSMS), is a rising technology in clinical laboratories for a variety of analyses [81,82,83,84,85,86,87,88,89]. LC-MS provides shorter runtimes than immunoassays, eases workflow, and is significantly lower in cost than other instrumental techniques [82,83,84,85]. Allison et al. (2021) described that mass spectrometry could be utilized as a screening method for fungal infections like IPA in clinical settings [90]. The method utilizes acid degradation of chitin, a fungal cell wall component, to its detectable low-molecular-weight products [90]. One of these products is glucosamine, a commonly studied amino monosaccharide [91,92,93]. Due to its low molecular weight and abundance in samples of Aspergillus, glucosamine has the potential to serve as a biomarker for Aspergillus infections [90,94]. The use of glucosamine as a fungal infection biomarker has not been tested on clinical samples. Other methods using LC-MS for Aspergillus detection target other biomarkers. Triacetylfusarine C, a fungal siderophore, has been detected in urine and serum in IPA-positive patients, and gliotoxin has been detected in serum samples collected from IPA-positive patients [95,96,97]. LC-MS is a rising technology in clinical laboratories; thus, these methods have great potential for new diagnostic standards in medical labs [82]. However, none of these methods has been evaluated clinically, so more studies are needed to validate and verify their clinical efficacy.

One specific mass spectrometry method being utilized in research studies for the detection of IPA is matrix-assisted laser desorption ionization–time-of-flight mass spectrometry (MALDI-TOF MS). MALDI-TOF MS is often used to identify microbes by comparing a characteristic spectrum of an unknown called a peptide mass fingerprint (PMF) to a database. MALDI-TOF MS is now being used to identify *Aspergillus* isolates in both solid media culture and blood culture [17,98,99,100]. *A. fumigatus*, along with other pathogenic species of *Aspergillus* (such as *A. niger*, *A. flavus*, *A. nidulans*, and *A. terreus*), and nonpathogenic species were able to be identified to the species level from clinical isolates. Using an in-house mold database generated from 53 *Aspergillus* species (and later provided to the MALDI-TOF MS manufacturer used in the study), 95.5% of samples were identified at the species level [98]. With the use of PMF databases, MALDI-TOF MS is also being used to identify distinct species and strains of *Aspergillus* from solid culture and blood culture, with 98.4% of samples being properly identified by the technique [99]. Methods have also been described that use MALDI-TOF MS to detect antifungal-medication-resistant strains of *Aspergillus fumigatus* [100].

While mass spectrometric methods are promising for clinical applications, there are limitations that could affect their clinical viability. First, and possibly most importantly, very few studies have been performed using LC-MS or MALDI-TOF MS on clinical samples. The lack of clinical studies using mass spectrometric methods hinders their practical use for diagnosis of IPA. Second, the majority of methods being developed for clinical applications use standard benchtop LC-MS systems, which are highly complex instruments. Benchtop LC-MS systems are large, have a high startup cost, and require technical specialists to operate and maintain them. LC-MS results can also vary depending on the cleanliness of the instrument and its internal components, the sample vessel, and various matrix effects [82,83,101]. Matrix effects are defined as changes in signal due to components of a sample that are not the analyte and are very common in LC-MS methods [102]. Due to these matrix effects, a robustness in method development and validation prior to the rollout of a clinical method are required, causing a high barrier to entry for new methods [101,102,103]. LC-MS is also not currently an automated platform, and, when compared to other assays, such as qPCR, ELISA, and PNA-FISH, has a high complexity for a relatively moderate-to-low throughput. Once LC-MS methods are eventually implemented for clinical use, they tend to boast low analytical detection limits, especially when using LC-MSMS systems, high analytical specificity and analytical sensitivity, and low relative cost [81,82,83,84,85,98,99,100,101,103,104,105,106].

## 3. Conclusions

Infections like IPA have been described since ancient times, and, in the past 20 years, there has been a push for novel, more sensitive diagnostic methods. Currently, culture, histopathology, and imaging are the most common methods for detection of IPA. With these methods, there are many variables that largely impact the accuracy of diagnosis, including what specimens are cultured, the method of specimen collection, culture media, and stage of infection. To mitigate some of these variables, decrease the invasiveness of some specimen collections, and decrease time to diagnosis, newer promising detection techniques have been developed. While PCR is the most studied alternate detection method, PNA-FISH, ELISA, lateral flow assays, and LC-MS all have shown promise as sensitive and standardizable techniques in the past 15 years. Compared to culture alone, all the described techniques also show a decrease in diagnosis time, increasing the odds of survivability from infections like IPA.

With all of the current detection methods in mind, continued investigation into diagnostic assay development for detecting *Aspergillus* is warranted for human patients. Further clinical studies into newer research laboratory techniques are warranted to test their clinical viability, sensitivity, and selectivity. Continued investigation will lead to both a greater understanding of *Aspergillus*-related diseases and lower mortality rates from those diseases.

## Figures and Tables

**Table 1:** Sensitivity and specificity of *Aspergillus* PCR assays [50].

Author	Sample Type	Clinical Sensitivity	Clinical Specificity
Tuon [53]	BAL fluid	78.4%	93.7%
Sun [54]	BAL fluid	79.6%	94.1%
Avni [55]	BAL fluid	76.8%	94.5%
Mengoli [56]	Blood	88.0%	75.0%
Arvanitis [57]	Blood	84.0%	76.0%

**Table 2:** Selectivity and sensitivity of several LFAs published in the literature.

Company	Sample Type	Clinical Sensitivity	Clinical Specificity
OLM Diagnostics *Asp*LFD	Serum [75]	68%	87%
	BAL fluid [32,75]	86–88%	93–95%
IMMY sona *Aspergillus* galactomannan LFD	Serum [76,77,78]	50–97%	83–92%
	BAL fluid [79]	77–92%	97–98%
Aspergillus ICT	Serum [80]	67.6%	81%

## Data Availability

Not applicable.
